# Staining and resin embedding of whole *Daphnia magna* samples for micro-CT imaging enabling 3D visualization of cells, tissues, and organs

**DOI:** 10.1371/journal.pone.0313389

**Published:** 2024-11-08

**Authors:** Mee S. Ngu, Daniel J. Vanselow, Andrew L. Sugarman, Rachelle A. Saint-Fort, Carolyn R. Zaino, Maksim A. Yakovlev, Keith C. Cheng, Khai C. Ang

**Affiliations:** 1 Department of Pathology, Pennsylvania State University College of Medicine, Hershey, Pennsylvania, United States of America; 2 Jake Gittlen Laboratories for Cancer Research, Pennsylvania State University College of Medicine, Hershey, Pennsylvania, United States of America; 3 Institute for Computational and Data Sciences, Pennsylvania State University, State College, Pennsylvania, United States of America; 4 Molecular and Precision Medicine Program, Pennsylvania State University College of Medicine, Hershey, Pennsylvania, United States of America; University of the Punjab Quaid-i-Azam Campus: University of the Punjab, PAKISTAN

## Abstract

Micro-CT imaging is a powerful tool for generating high-resolution, isotropic, three-dimensional datasets of whole, centimeter-scale model organisms. At histological resolutions, micro-CT can be used for whole-animal qualitative and quantitative characterization of tissue and organismal structure in health and disease. The small size, global freshwater distribution, wide range of cell size and structures of micron scale, and common use of *Daphnia magna* in toxicological and environmental studies make it an ideal model for demonstrating the potential power of micro-CT-enabled whole-organism phenotyping. This protocol details the steps involved in *D*. *magna* samples preparation for micro-CT, including euthanasia, fixation, staining, and resin embedding. Micro-CT reconstructions of samples imaged using synchrotron micro-CT reveal histological (microanatomic) features of organ systems, tissues, and cells in the context of the entire organism at sub-micron resolution and in 3D. The enabled “3D histology” and 3D renderings can be used for morphometric analyses across cells, tissues, and organ systems.

## Introduction

Micro-computed tomography (micro-CT) is becoming recognized as a tool for three-dimensional (3D) visualization and quantitative analysis of biological samples. Imaging whole, intact samples allows detailed investigation of overall morphology and cellular structures and is especially useful for evaluating microanatomy and phenotypes in various model organisms [1–5]. *Daphnia magna* is a keystone branchiopod crustacean (order Cladocera) in lentic ecosystems worldwide and an established model in ecology and evolution [6, 7]. They have a primarily parthenogenetic life cycle that produces clonal populations from single genotypes. Their short generation time permits the experimental manipulation of large populations for concurrent studies of molecular and phenotypic responses to stressors. They are commonly used for their responses to environmental stressors [8–11] and toxins [12–15], and as a complementary model for other animal models in higher taxa. Using histotomography, micro-CT optimized for cellular characterization [2], we imaged adult *D*. *magna* using wider-field lens and camera instrumentation capable of 0.5-micron isotropic resolution [16]. Here, we share details of sample preparation used in that work that is reproducible for micro-CT on commercial instrumentation.

Sample preparation that results in well-preserved microanatomical features is the first step in generating high-quality micro-CT images. This protocol includes details of the sample preparation of whole *D*. *magna* for micro-CT imaging, including euthanasia, fixation, staining with metal, and resin embedding. Metal staining enhances otherwise weak inherent contrast between soft tissues in micro-CT images. Phosphotungstic acid (PTA), a heteropoly acid with the chemical formula H₃PW₁₂O₄₀, is one of the most widely used single contrast agent for micro-CT imaging because it provides superior contrast between tissue components [17]. The problems with PTA staining include length of staining and uniformity. For example, PTA staining of invertebrates can take up to one week or longer [18–21]. The commonly used PTA concentration of 0.3% works for staining small juveniles (instar 1–3 or 1–3 days after extruding from brood chamber) within 48 hours but is associated with inconsistent staining in gravid adults (instar 8 and older, S1 Fig). A higher concentration of PTA (3%) provides consistent staining of the whole *D*. *magna* adults in three days. Samples can be kept in ethanol if they can be scanned immediately. If immediate imaging is not needed or available, sample dehydration and embedding in resin [22] is recommended because samples stored in ethanol deteriorate over time. The scope and scale of images made possible by the protocol provided is a necessary step towards enabling computational morphological analysis of genetic and environmental change in *Daphnia* and other Cladocera species.

## Materials and methods

The protocol described here is published on protocol.io, DOI: dx.doi.org/10.17504/protocols.io.6qpvr8dwolmk/v1, and is included for printing as supporting information S1 File with this article.

### Daphnia magna culturing

A commercial strain of *D*. *magna* was purchased from Carolina Biological (NC, USA) and raised in “Aachener Daphnien-Medium” or ADaM at room temperature (20°C ± 1°C) under a 16-hour light/8-hour dark photoperiod. *D*. *magna* cultures were fed three times weekly with 3.0 x 10^7^ cells/ml of green microalgae (*Raphidocelis subcapitata)* and 0.1 mg/mL of dissolved bakers’ yeast once a week.

### Euthanasia, fixation, and staining

*D*. *magna* samples were euthanized in carbonated water and fixed in Bouin’s solution for 24 h. After fixation, samples were dehydrated with 35%, 50%, and 70% ethanol for 15 min each, at room temperature, with agitation before staining in PTA for at least 48 h, depending on the size or age of the samples. After staining, samples can be stored and imaged in 70% ethanol within three days. Further serial dehydration using 90%, 95%, and 100% ethanol, for 20 min each, at room temperature with gentle agitation, followed by overnight resin infiltration and embedding of samples in LR White for immediate imaging, long-term storage, and data re-acquisition.

### Micro-CT imaging and reconstruction

Scans were first performed on an in-house custom benchtop system at lower resolution to confirm staining, then at synchrotron beamline 8.3.2 at the Advanced Light Source at Lawrence Berkeley National Laboratory for final scans. The custom benchtop micro-CT system utilized an Indium Gallium liquid metal jet X-ray source (Excillum D2+) with a LuAG (Metal-laser) scintillator and a 10mm field-of-view/0.7 um pixel size imaging system. Source anode voltage was set to 70kV and 150W and 500 rotation projections were taken per scan. Exposure time per projection was 1200 ms. Samples were rotated with continuous motion over 220 degrees during each imaging session. Source to sample distance was 208 mm. The source-to-scintillator distance was set to 19 mm to avoid using cone-beam reconstruction. The camera (Vieworks VP-151MC) was set to hardware SUM bin4 to boost the signal. Reconstructions were performed using parallel geometry with the gridrec algorithm in Tomopy [23]. The final image voxel size was 2.8 μm^3^.

Synchrotron scans were acquired using a 5 mm field-of-view/0.5 μm pixel resolution imaging system at 20 keV as a sequence of 150 ms projections [16]. About 3000 projections were obtained over 180° for adult females. Additionally, 20 flat-field (gain) images (at the beginning and end of acquisition) and 20 dark-field images were also acquired. Flat-field correction, stripe removal, and image reconstruction were performed using the open-source TomoPy toolkit [23]. Reconstructions resulted in an isotropic voxel size of 0.52 μm^3^.

## Results

**Fig 1:**
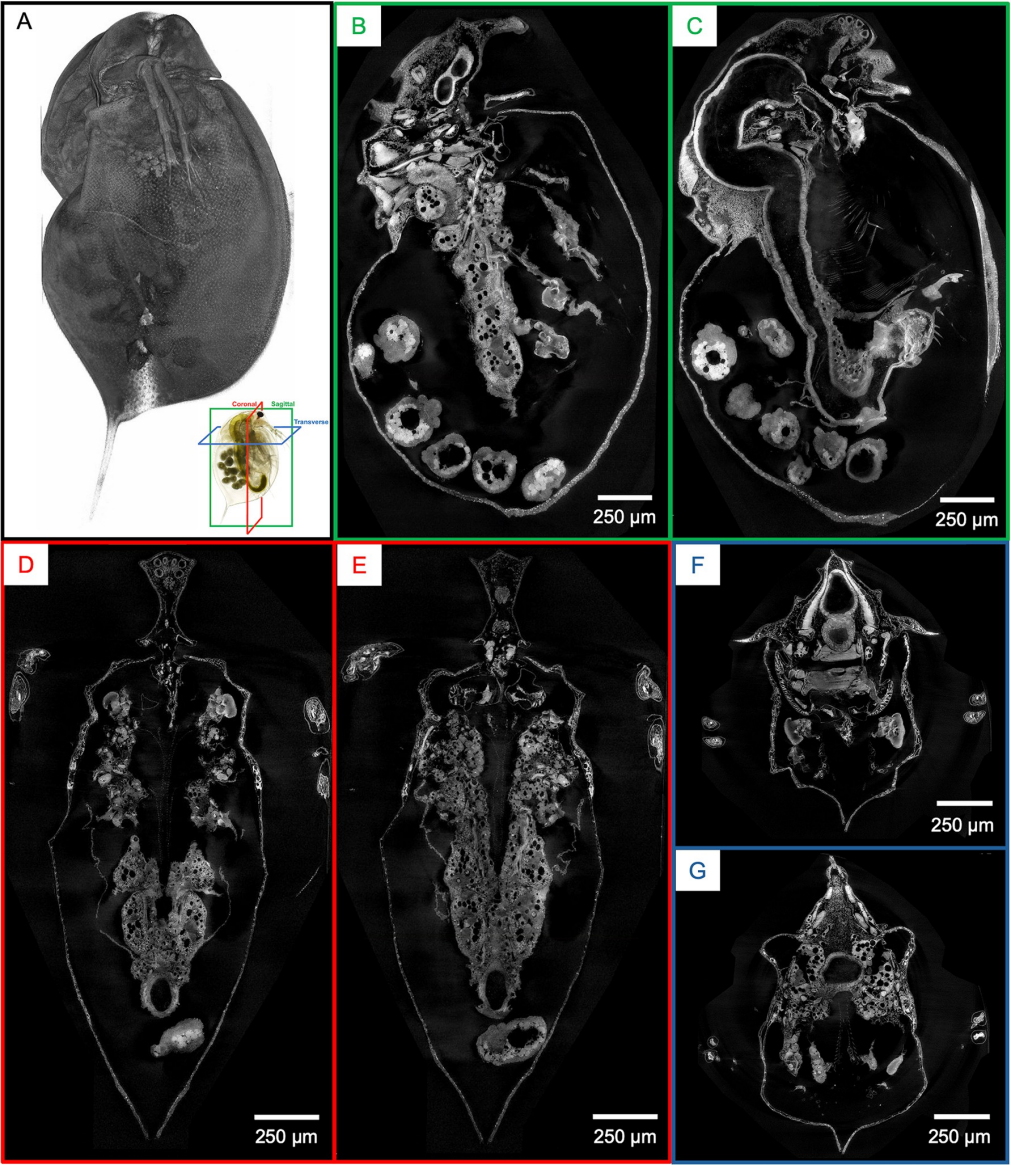
Whole-organism imaging of PTA-stained *D*. *magna* at cell resolution enables histology-like cross sections. (A) 3D volume rendering shows the scanning electron microscopy-like surface rendering. Sagittal (B, C), coronal (D, E), and transverse (F and G) cross-sections can be obtained from one sample after imaging. B to G represent 5 μm thick micro-CT slabs.

**Fig 2:**
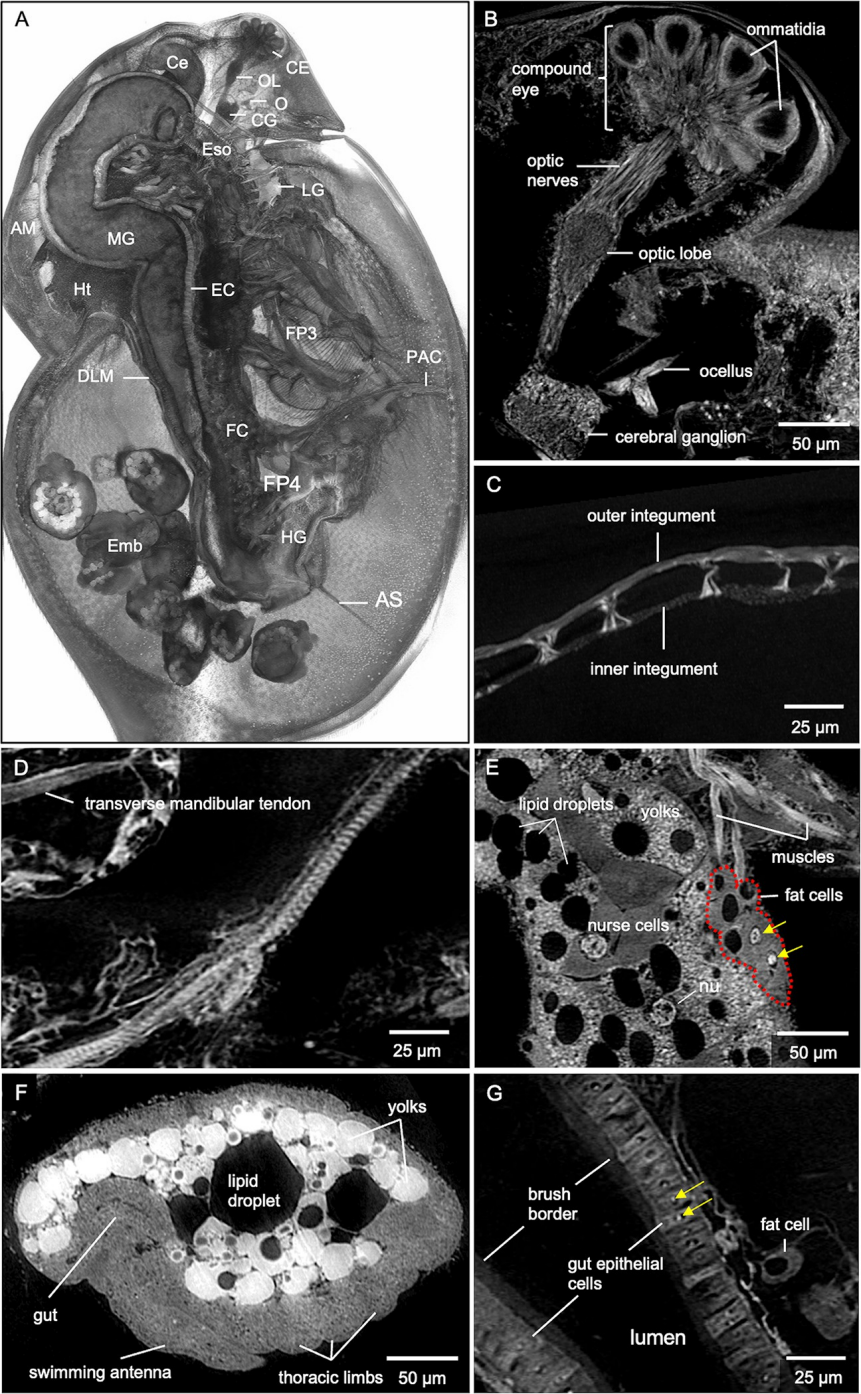
Microanatomic features of an adult female *D*. *magna* from synchrotron-based micro-CT imaging at 0.5 μm per pixel resolution. (A) 3D rendering at the mid-section of the sagittal plane with various organs, organ substructures, and cell types indicated. AM, antennal muscles; AS, abdominal setae; Ce, hepatic ceca; CE, compound eye; CG, cerebral ganglia; DLM, dorsal longitudinal muscles; EC, gut epithelial cells; Emb, developing embryos; Eso, esophagus; FC, fat cells; FP3, filter plates on third pair of thoracic limbs; FP4, filter plates on fourth pair of thoracic limbs; HG, hindgut; Ht, heart; LG, labral glands; MG, midgut; O, ocellus, OL, optic lobe; PAC, post-abdomen claws. Highlights of microanatomical features include (B) ommatidia of the compound eye, optic nerves, optic lobe, cerebral ganglia, and ocellus. Other features include (C) filamentous actin bundle pillars between inner and outer carapace integuments and (D) muscle striations along the thoracic muscle. (E) Details in the ovary include nucleus (nu) of the oocyte, nurse cells, yolks, and lipid droplets. Nucleoli (yellow arrows) in fat cells are also clearly visible. (F) Recognizable details in the developing embryo include precursors of the gut, swimming antennae, and thoracic limbs. (G) The brush border and epithelial cells with nucleoli (yellow arrows) within the nuclei are visible in the gut. B represents a 5 μm thick micro-CT slab; C represents a 5μm thick micro-CT slab; D to G represent individual 0.5 μm thick micro-CT slices.

The protocol described here details sample preparation of whole *Daphnia magna* for micro-CT imaging. Resulting 3D reconstructions reveal anatomic (organ) and micro-anatomic (cellular) features in the context of the entire organism. Preliminary micro-CT reconstructions at 2.8 μm voxel resolution allow visualization of various organs (S2 Fig). Synchrotron-based micro-CT whole-organism reconstructions at 0.5 μm resolution add cellular detail within each organ. The similarity of digital slices to conventional histology output is demonstrated by representative coronal, sagittal, and transverse cross-sections (Fig 1). Highlights include the ommatidia (22 in total) of the compound eye, optic nerves (22 in total), and cellular details in the optic lobe and cerebrum ganglia (Fig 2B). Filamentous actin bundle pillars between carapace integuments (Fig 2C) and striations on the muscle of the thoracic limb (Fig 2D) can easily be seen. Features in the ovary include yolks, lipid droplets, nurse cells, and the nucleus of an egg (Fig 2E). Fat cells around the ovary consist of one or several lipid droplets and one large nucleus (about 10 μm in diameter) within which a nucleolus of irregular shape resides (Fig 2E). Development of the embryos can be observed according to the size and shape of the gut, swimming antenna, and thoracic limb precursors (Fig 2F). In the gut, a single epithelial layer is seen with epithelial cells containing nucleoli that measure about 2.5 μm in diameter (Fig 2G). Individual intestinal microvilli cannot be visualized because they are smaller than 1 micron in diameter. However, their collective directionality, perpendicular to the gut surface, is represented by the texture of a brush border that is about 7 μm in height (Fig 2G).

**Fig 3:**
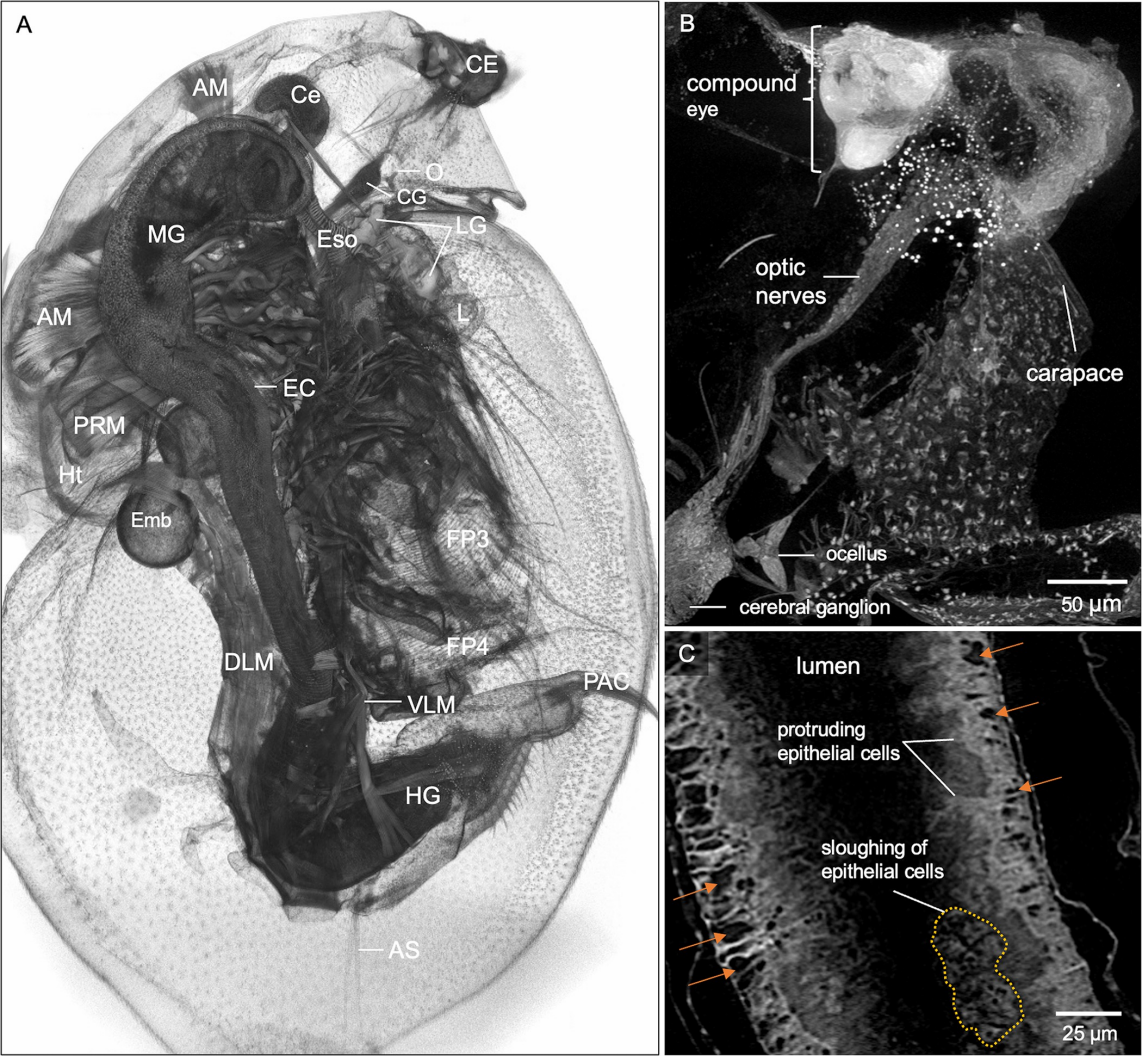
Microanatomic features of a wild-type female *D*. *magna* with an atypical eye. (A) 3D rendering at the mid-section of the sagittal plane with various organs and organ substructures indicated. AM, antennal muscles; AS, abdominal setae; Ce, hepatic ceca; CE, compound eye; CG, cerebral ganglia; DLM, dorsal longitudinal muscles; EC, gut epithelial cells; Emb, developing embryos; Eso, esophagus; FP3, filter plates on third pair of thoracic limbs; HG, hindgut; Ht, heart; L, labrum; LG, labral glands; MG, midgut; O, ocellus; PAC, post-abdomen claws. (B) Micro-CT provides details where the compound eye (CE) has less than 22 ommatidia with irregular shape and arrangement. The optic nerves (ON) connect directly to the cerebral ganglia without an optic lobe. (C) Whole organism micro-CT imaging also revealed abnormalities in the gut, with an excessive number of epithelial gaps (orange arrows), protruding and sloughing of the gut epithelial cells. B and C represent 25 and 5 μm thick micro-CT slabs, respectively.

The ability to interrogate the cellular and organ-based structures throughout the entire organism is essential for characterizing whole-organism tissue phenotyping. To exemplify the detection of morphological change, we image a wild-type female *D*. *magna* with an atypical eye (Fig 3). Micro-CT imaging provides details of the abnormal compound eye, which contains less than the usual 22 ommatidia, with irregular shape and arrangement (Fig 3B). The optic nerves are longer and connect directly to the cerebral ganglia without an optic lobe (Fig 3B). Moreover, abnormalities are detected in the gut where there are excessive epithelial gaps, protruding, and sloughing of gut epithelial cells (Fig 3C), which would potentially go unnoticed using an imaging technology that does not consider the entire specimen at cellular detail. Side-by-side comparison of the normal wild-type *D*. *magna* versus wild-type with atypical eye is presented in supplementary figure (S3 Fig).

**Fig 4:**
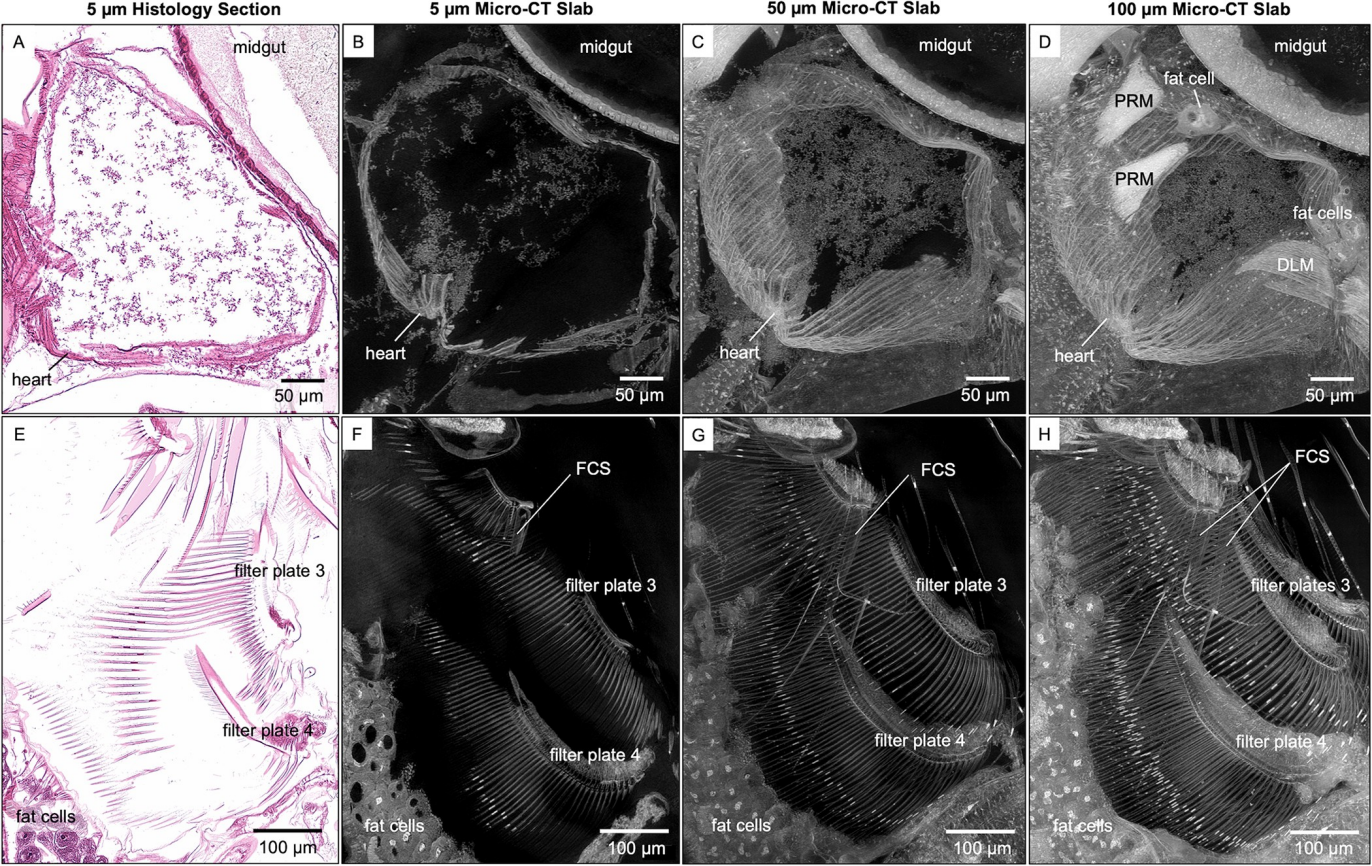
Visualization of anatomical structures using micro-CT slabs of various thicknesses. The 5 μm thick micro-CT slab of heart and filter plates (B and F, respectively) resembles the 5 μm thick histological tissue section (A and E, respectively). Thicker micro-CT slabs (50 μm) allow visualization of (C) more heart wall muscles and (G) overlapping long setae of the filter plates. Micro-CT slabs of 100 μm showed (D) the posterior rotator muscles of the mandible (PRM) and fat cells around the heart, (H) both filter-cleaning spines (FCS) on the second pair of thoracic limbs, and both filter plates on the third pair of thoracic limbs (H). DLM, dorsal longitudinal muscles.

**Fig 5:**
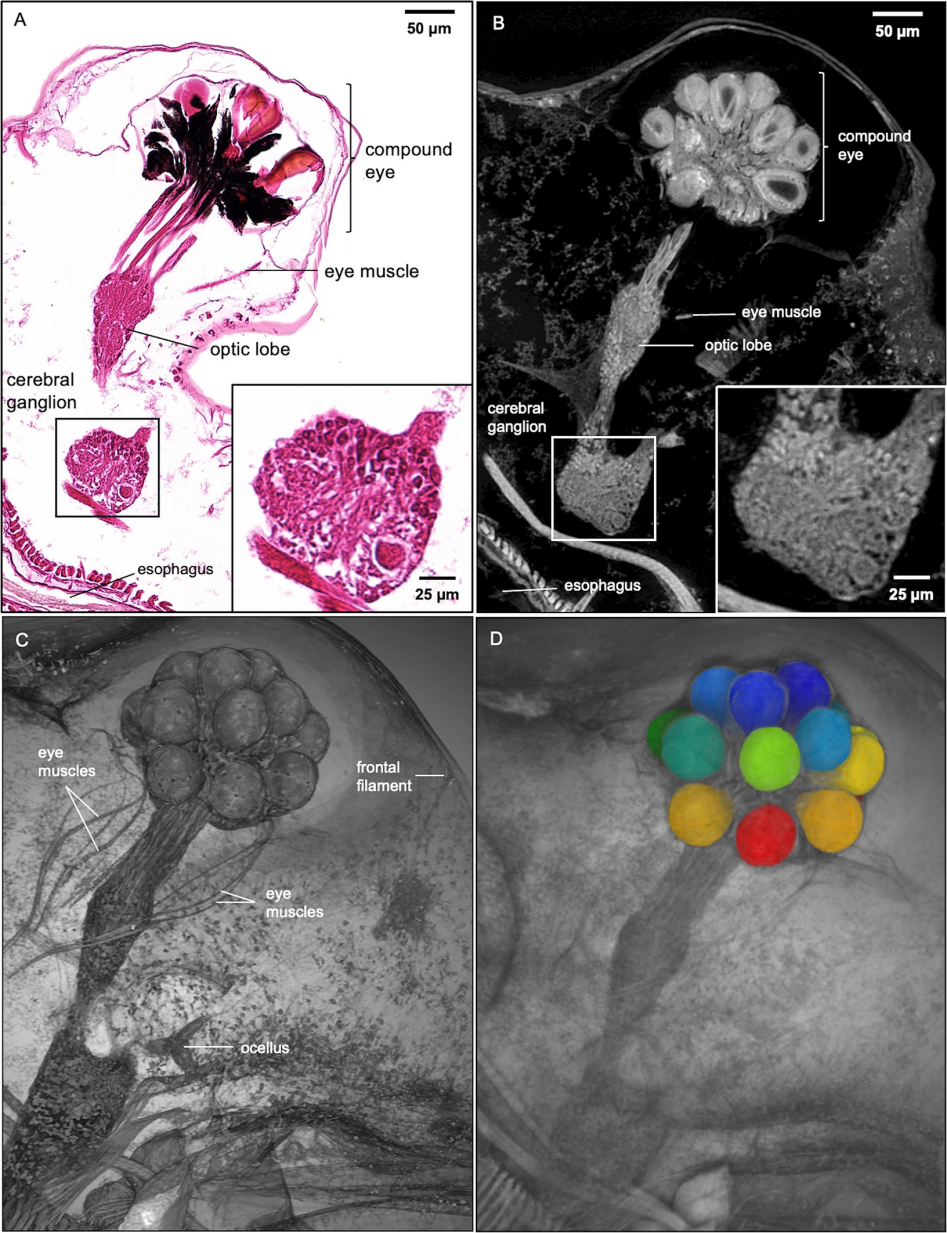
Histology section, micro-CT image, and 3D Rendering of *D*. *magna* vision system. Comparison of *D*. *magna* visual system using 5 μm thick histology section and micro-CT slab of the same thickness (A and B, respectively). Insets show details of cerebral ganglion where the micro-CT slab demonstrates the near histological resolution of micro-CT imaging at 0.5-micron resolution. (C) 3D rendering featuring the vision system allows clear visibility of the frontal filament that connects to the ocellus, the eye muscle bundles, and their insertion into the compound eye. (D) Image segmentation of structures of interest (crystalline cones shown here) allows the isolation of specific structures for measurement or quantitative analysis.

While both traditional histology and micro-CT imaging have the resolution needed to distinguish cellular features in 2D slices, only the latter can reveal thicker, complex 3D tissue structures such as heart and paired filter plates on the third and fourth pairs of thoracic limbs (Fig 4). Slabs are generated from the maximum intensity projection of micro-CT slices. Customizing micro-CT slab thicknesses for specific anatomical structures enable visualization of whole organs in detail and with spatial context. Besides seeing more heart muscles using thicker slabs, posterior rotator muscles of the mandibles and fat cells around the heart can also be seen (Fig 4D). Filter-cleaning spines (FCS, on the second pair of thoracic limbs) that bear uniseriate rows of widely spaced spinules become apparent in thicker micro-CT slabs (Fig 4G and 4H). The overlapping of long setae on the filter plates with the filter-cleaning spines is also apparent (Fig 4G and 4H). Besides slabs, visualization of multiple anatomical structures, such as the compound eye, optic nerves, optic lobe, cerebral ganglia, and ocellus in the vision system, can also be customized using 3D rendering with various viewing angles and cutting planes (Fig 5). The insertion of eye muscles to the compound eye and the connection of frontal filaments to ocellus can be easily visualized using 3D rendering (Fig 5C).

## Discussion

In developing this protocol, we prioritized time efficiency and sufficient detail for newcomers to prepare whole *Daphnia* samples for micro-CT imaging. Bouin’s is the fixative of choice for whole *Daphnia* samples because paraformaldehyde and 10% neutral buffered formalin yield less consistent fixation. Samples fixed overnight in 4% paraformaldehyde and 10% neutral buffered formalin tend to exhibit a fixation artifact in which the carapace is expanded, and the post-abdomen is extended ventrally, causing embryos in gravid *Daphnia* to be dislodged from the brood chamber [24]. Fixation of several arthropod taxa in Bouin’s has also been reported to provide better results in terms of tissue contrast when compared with ethanol and glutaraldehyde solution [25].

PTA stain is commonly used at concentrations of 0.3–0.5% for micro-CT imaging (17,22). While the lower concentration of 0.3% PTA is sufficient for small/juvenile *D*. *magna*, it does not provide homogenous staining of gravid adults after 72 hours. In contrast, 3% PTA provides consistent staining of gravid adult samples in 72 hours, resulting in an ideal contrast for high-resolution micro-CT imaging. For adult samples carrying many developing embryos (>15), an additional 24 hours is needed to ensure that all the embryos are stained completely. Renewal of PTA solution after 48 hours of incubation is important for achieving homogenous staining. The optimal PTA concentrations and time-efficient staining durations to achieve even contrast for samples of various ages are summarized in Table 1.

**Table 1:** Concentration of PTA and staining duration for different Ages of *D*. *magna*.

Age	Embryos in brood chamber	PTA concentration	Staining duration
Juvenile (instar^a^ 1–3)	no	0.3%	48 hrs
Juvenile (instar^a^ 4–7)	no	1%	48 hrs
Adult (instar^b^ 8 and older)	yes	3%	72 hrs or longer (with a PTA renewal every subsequent 48 hrs)

^a^One instar is about 24 hours for juveniles.

^b^One instar is about 72 hours for adults.

Besides 70% ethanol, agarose is another embedding medium for immediate imaging or short-term storage [26]. From our experience, resin-embedded *Daphnia* samples are suitable for immediate imaging, long-term storage of samples, and data re-acquisition. A degree of sample distortion or shrinkage in the carapace and internal organs is apparent from the visualizations of the micro-CT reconstructions. Such distortion is common to fixation methods involving dehydration, including histology [27] and critical point drying technique used for scanning electron microscopy [25]. Laforsch and Tollrian (2000) demonstrated that drying by hexamethyldisilazane yielded better surface structure than critical point drying technique [28]. However, without solid supportive matrix, sample mounting for micro-CT imaging might be challenging and result in sample damage. Without a solid supportive matrix, any movement (especially the swimming antennae) during micro-CT imaging might result in motion artifacts that will yield blurry and unusable images. Nonetheless, readers are encouraged to consider all the options, and development of methods that are not associated with distortion would be a valuable subject of future work.

The above protocol designed for micro-CT imaging of *Daphnia* is applicable to other Cladocera. It may be adaptable to other chitinous terrestrial invertebrates of similar size for broader taxonomic, ecological, anatomic, genetic, and toxicological studies.

## Supporting information

S1 File Step-by-step protocol, also available on protocol.io. (PDF)

S1 Fig Uneven staining of adult gravid *D*. *magna*. Only some muscles and epipodites of the thoracic limbs, a portion of the developing embryos, and carapace were stained in 0.3% PTA after 48h.
(TIFF)

S2 Fig Anatomic features of an adult female shown by scan from benchtop micro-CT scanner at 2.8 μm per pixel resolution. AM, antennal muscles; Ce, hepatic ceca; CE, compound eye; CG, cerebral ganglia; Cp, carapace; DLM, dorsal longitudinal muscles; Emb, developing embryos; Ht, heart; LG, labral glands; MG, midgut; O, ocellus; OL, optic lobe; Ov, ovary; TL, thoracic limbs.
(TIFF)

S3 Fig Side-by-side comparison of the normal wild-type *D*. *magna* versus wild-type with atypical eye. 3D rendering of (A) a normal wild-type *D*. *magna* versus (B) a wild-type *D*. *magna* with an atypical eye. (C) Normal compound eye with optic nerves connect to optic lobe and cerebral ganglia. (D) Abnormal compound eye protruded from the carapace with irregular shape and arrangement of the ommatidia. The optic lobe is absent in this *D*. *magna*. (E) Single layer of gut epithelial cells in the wild-type *D*. *magna* versus (F) gut lining with protruding and sloughing of epithelial cells in *D*. *magna* with atypical eye.
(TIFF)
