# Supplementary material for: Staining and resin embedding of whole Daphnia magna samples for micro-CT imaging enabling 3D visualization of cells, tissues, and organs
Source: PLoS One. 2024 Nov 8;19(11):e0313389. doi: 10.1371/journal.pone.0313389 (PMC11548835; doi:10.1371/journal.pone.0313389)
Supplement: S1 File — (PDF) [file pone.0313389.s001.pdf]

Staining and resin embedding of whole *Daphnia magna* samples for micro-CT imaging enabling 3D visualization of cells, tissues, and organs

Mee S. Ngu, Daniel J. Vanselow, Andrew L. Sugarman, Rachelle A. Saint-Fort, Carolyn R. Zaino, Maksim A. Yakovlev, Keith C. Cheng, Khai C. Ang

Euthanasia

1d

1 Transfer the *Daphnia* sample using bulb pipette containing the least amount of water into a flat bottom glass vial such as a 20 mL scintillator vial (Wheaton) filled with carbonated water to euthanize the *Daphnia*. As soon as the sample stops moving, transfer it into Bouin's solution.

Note

Tip of bulb pipette trimmed at a 45° angle such that the diameter is bigger than the size of the sample.
Sample should be transferred individually to prevent damaging the sample.

Fixation

1d

2 Immediately, replace the Bouin's solution and fix the sample in fresh Bouin's solution at Room temperature for 24:00:00 (hrs).

1d

Note

Replacing with fresh Bouin's solution is to remove any water carried over.

Staining

1h 25m

3 Rinse the fixed sample with 1x phosphate buffered saline (PBS) pH 7.4 for 10 minutes (min), thrice.

3.1 Rinse the fixed sample with 1x PBS pH 7.4 for 00:10:00 (min) (1/3).

10m

3.2 Rinse the fixed sample with 1x PBS pH 7.4 for 00:10:00 (min) (2/3).

10m

3.3 Rinse the fixed sample with 1x PBS pH 7.4 for 00:10:00 (min) (3/3).

10m

4 Submerge the sample in 35% ethyl alcohol (EtOH) for 00:15:00 at 23 °C with gentle agitation.

15m

5 Discard the 35% EtOH and submerge the sample in 50% EtOH for 00:15:00 at Room temperature with gentle agitation.

15m

Note

Graded dehydration of 35%, 50% and then 70% is performed to prevent severe shrinkage artifact in *Daphnia* sample.

6 Discard the 50% EtOH and submerge sample in phosphotungstic acid (PTA) in 70% EtOH at Room temperature with gentle agitation. Concentration of PTA and staining duration depend on the sample's age (Table 1).

Note

Replacement of PTA stain solution is highly recommended after 48 hours, especially for samples with developing embryos in the brood chamber.

A	B	C	D
**Age**	**Embryos in brood chamber**	**PTA concentra tion**	**Staining duration**
Juvenile (instar 1-3)	no	0.3%	48 hrs
Juvenile (instar 4-7)	no	1%	48 hrs
Adult (instar 8 an d older)	yes	3%	72 hrs or longer (with a PTA ren ewal at 48 hrs)

Table 1. Concentration of PTA and staining duration for different ages of *D. magna*

7 After staining, wash the sample in 70% EtOH for 5 min with gentle agitation, twice.

7.1 After staining, wash the sample in 70% EtOH for 00:05:00 with gentle agitation (1/2). 5m

7.2 After staining, wash the sample in 70% EtOH for 00:05:00 with gentle agitation (2/2). 5m

8 For adult sample to be scanned in 70% EtOH, transfer the sample using a trimmed bulb pipette or 1 mL pipette tip into a 200 µL micropipette tip and seal the tip end with polymer oven-bake clay (Sculpey). For smaller juvenile samples, 10 µL micropipette tips can be used.

9 Tap the sealed end gently to release bubbles.

10 Use clean round tip forceps or wooden stick to GENTLY push the sample down the pipette tip until it is immobilized against the wall of the pipette tip, without squeezing or crushing it.

11 Seal the opening of the pipette tip with parafilm M to avoid evaporation and the sample is ready to be scanned (Fig 1).

Note

In general, PTA-stained samples can be stored up to a month. If samples do not need to be permanent, scanning *Daphnia* samples at this step is possible within 3 days without ballooning artifact. To avoid ballooning artifact and to allow imaging of *Daphnia* more than 3 days after preparation we recommend serial dehydration and resin-embedding (below).

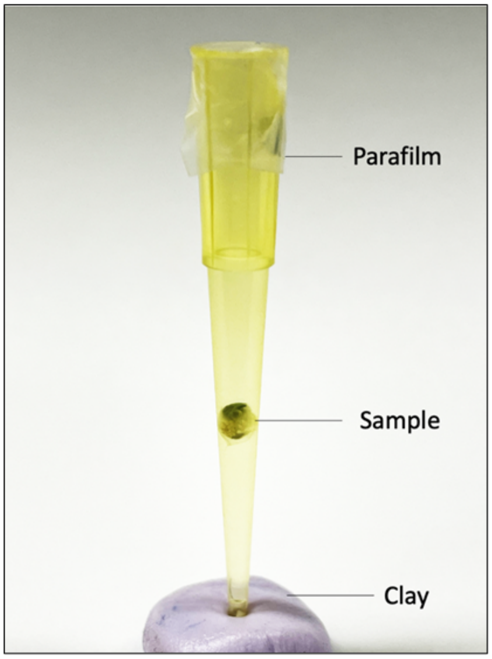

Fig 1. Gravid *D. magna* sample in 200 mL micropipette tip for imaging.

Dehydration and Resin Embedding (optional)

1d 6h 20m

12 For dehydration, submerge the sample in 90%, 95%, 100% and 100% EtOH for 20 minutes each concentration at Room temperature with gentle agitation.

Note

100% or 200 proof EtOH is the choice of dehydration agent for LR White acrylic resin according to LR White technical datasheet .

12.1 Submerge the sample in 90% EtOH for 00:20:00 . 20m

12.2 Submerge the sample in 95% EtOH for 00:20:00 . 20m

12.3 Submerge the sample in 100% EtOH for 00:20:00 . 20m

12.4 Submerge the sample in 100% EtOH for 00:20:00 .

20m

13 Prepare 1:1 v/v mixture of 100% EtOH and LR White acrylic resin. Submerge the samples in 1:1 EtOH and LR White acrylic resin mixture Overnight or at least 03:00:00 at Room temperature with gentle agitation.

3h

14 Submerge the sample in 100% LR White resin for 02:00:00 at Room temperature with gentle agitation.

2h

15 Replace the LR White resin with fresh 100% LR White resin and submerge for 01:00:00 at Room temperature with gentle agitation.

1h

16 Cut a polyimide tubing of appropriate diameter to 30 - 50 mm length (Table 2).

A	B	C
**Age**	**Polyimide tubing inner diameter**	**Micropipette tip**
Juvenile (instar 1-3)	0.0403”	200 mL (end of tip clipped off)
Juvenile (instar 4-7)	0.0808”	200 mL
Adult (instar 8 and older)	0.105”	1000 mL

Table 2. Sizes of polyimide tubing and micropipette tip for different ages of *D. magna*

17 Attach the polyimide tubing to a micropipette tip so that it fits snugly (Fig 2).

Note

200 µL micropipette tip for 0.0403” tubing and 1000 µL micropipette tip for 0.105” tubing. Clip off the end of 200 µL micropipette tip at 4.4 cm for the tubing to fit snugly.

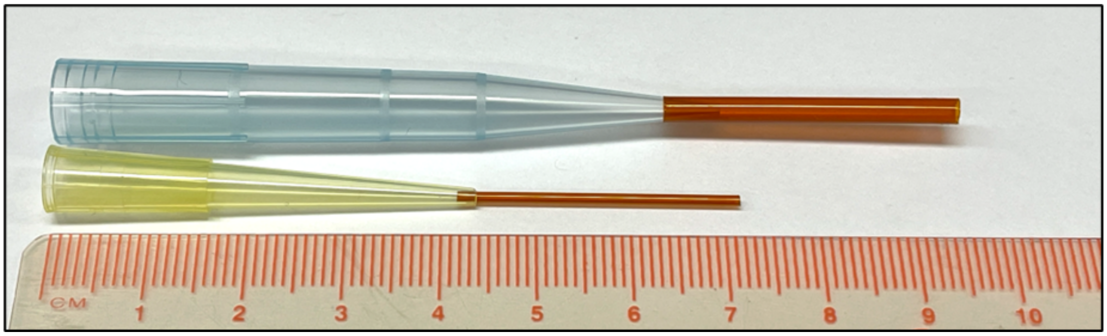

Fig 2. Polyimide tubing attached to micropipette tips for LR White resin embedding.

18 Attach the micropipette tip together with the polyimide tubing to a micropipette.

19 Transfer the sample to a small weigh boat or V-shaped solution basin and fully submerge the samples in fresh 100% LR White resin.

20 Position the tubing at the head of the sample and pipette resin to fill up half of the tubing before pipetting the specimen slowly into the tubing. Position the sample in the middle of the tubing and ensure the tubing above and below the tubing us filled with resin.

Note

Pipette the sample into the tubing such that the sample is moving in the natural, forward direction to avoid backward movement that will damage extremities.

21 Immediately seal the open end tightly, using a slab of oven-bake clay that has been flattened into a ~2mm-thick sheet. Remove excess clay.

22 Separate the micropipette tip from the polyimide tube by gentle rotation and seal that end of the tube with clay.

23 Place the tubing horizontally, with one end slightly elevated to minimize bubble formation next to the sample. Allow the resin to polymerize for 24:00:00 at 65 °C . Once polymerized, the sample is ready for imaging (Fig 3). Removal of polyimide tubing is possible but not necessary for imaging.

1d

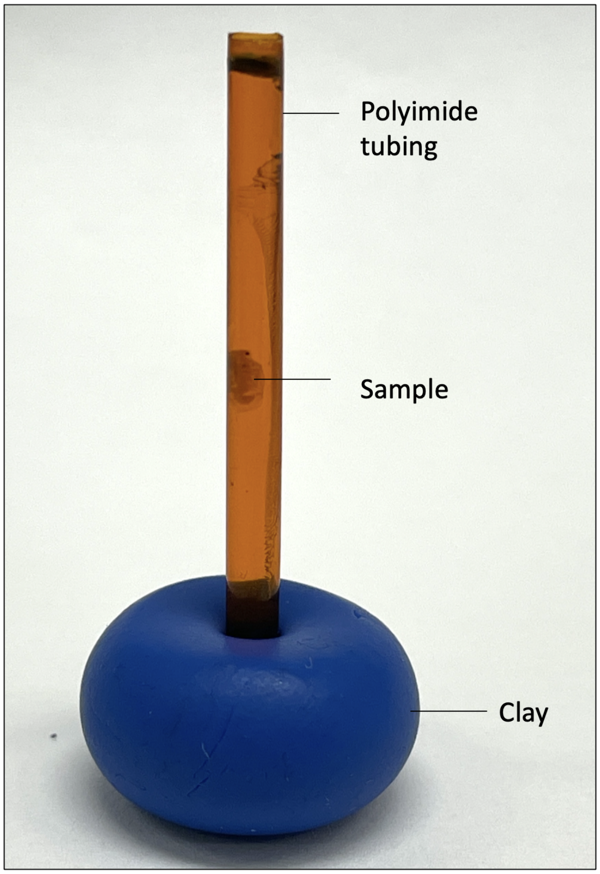

Fig 3. Sample embedded in LR White acrylic resin using polyimide tubing.
