# Supplementary figures and images for: Staining and resin embedding of whole Daphnia magna samples for micro-CT imaging enabling 3D visualization of cells, tissues, and organs
Source: PLoS One. 2024 Nov 8;19(11):e0313389. doi: 10.1371/journal.pone.0313389 (PMC11548835; doi:10.1371/journal.pone.0313389)

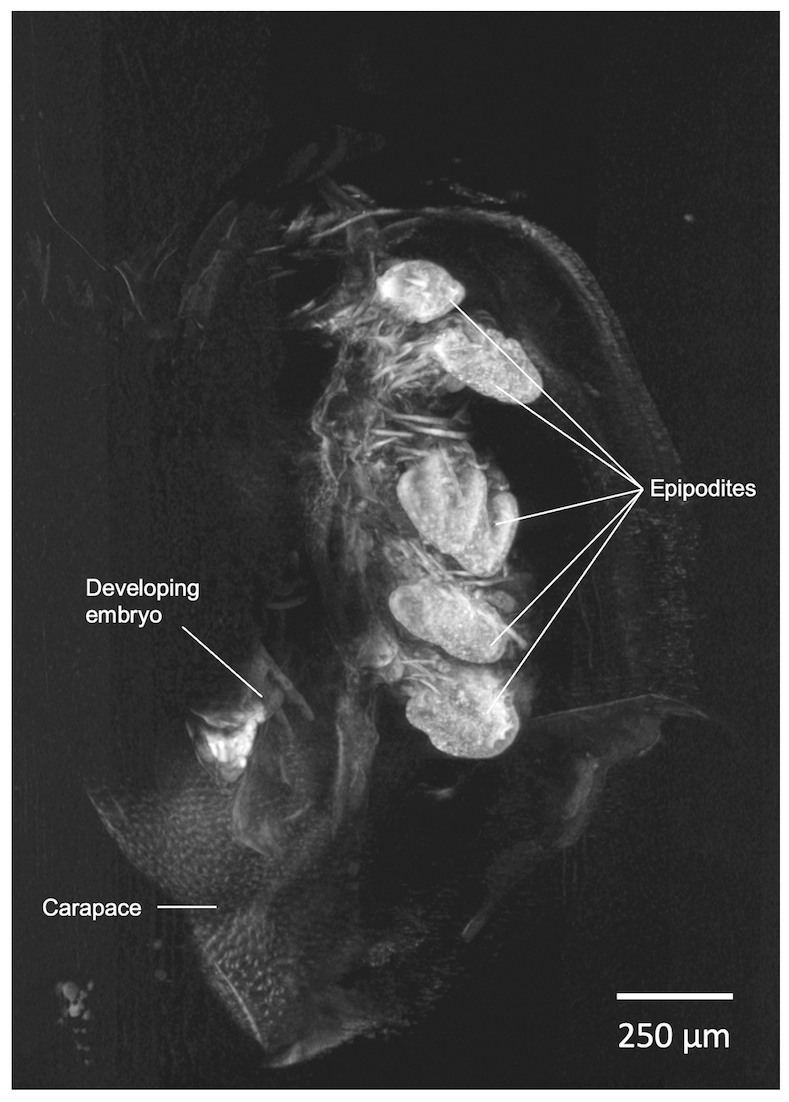

Supplement: S1 Fig — Only some muscles and epipodites of the thoracic limbs, a portion of the developing embryos, and carapace were stained in 0.3% PTA after 48h. (TIFF) [file pone.0313389.s002.tiff]

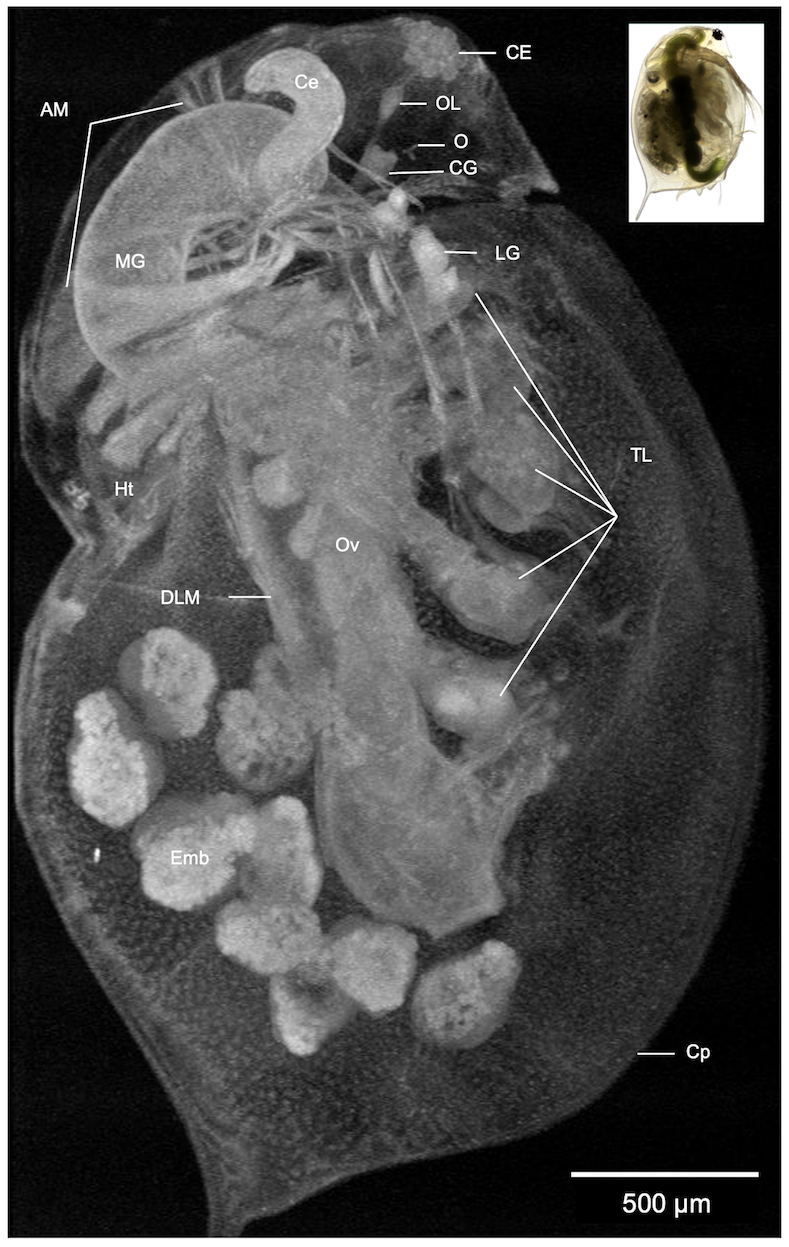

Supplement: S2 Fig — AM, antennal muscles; Ce, hepatic ceca; CE, compound eye; CG, cerebral ganglia; Cp, carapace; DLM, dorsal longitudinal muscles; Emb, developing embryos; Ht, heart; LG, labral glands; MG, midgut; O, ocellus; OL, optic lobe; Ov, ovary; TL, thoracic limbs. (TIFF) [file pone.0313389.s003.tiff]

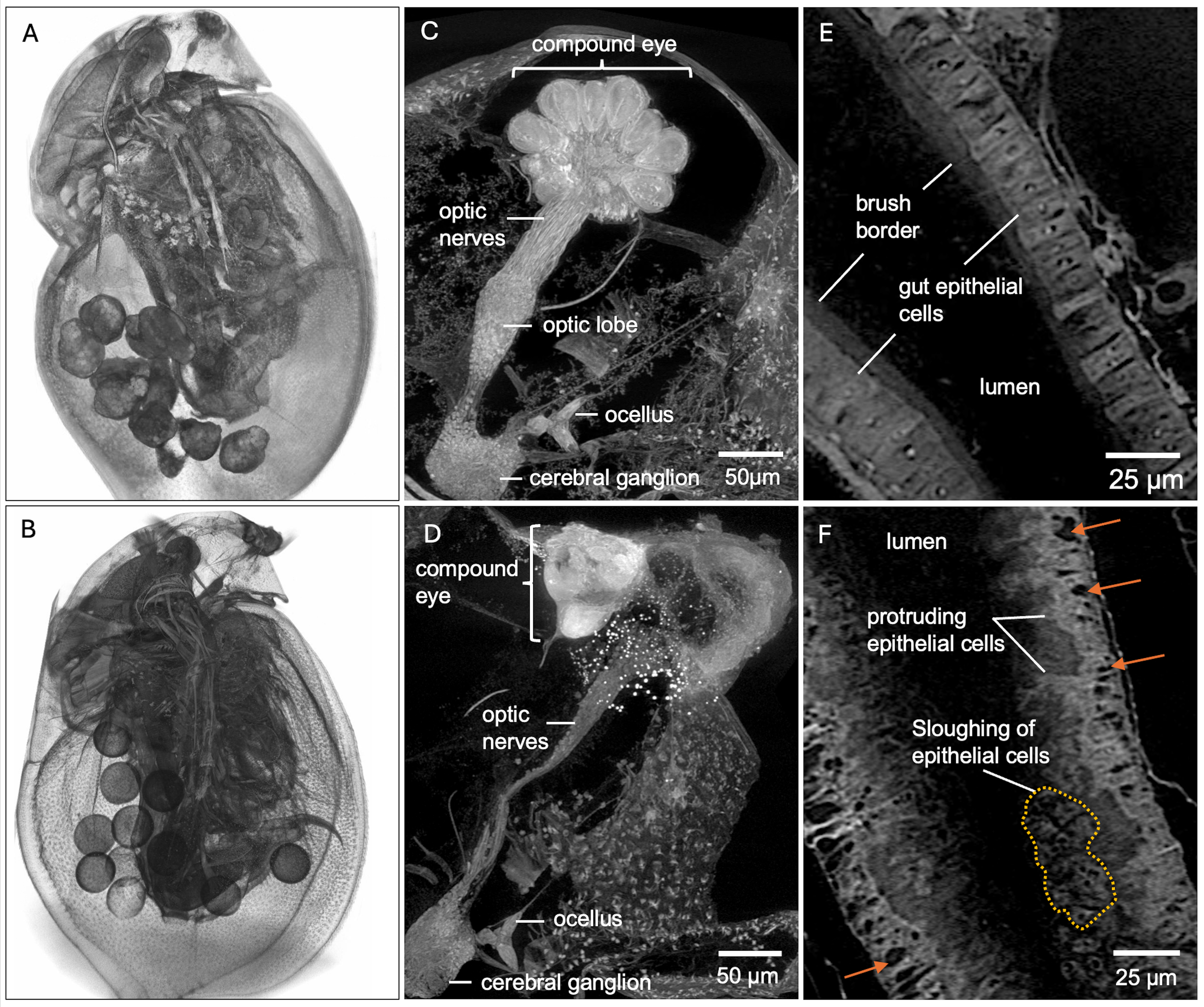

Supplement: S3 Fig — 3D rendering of (A) a normal wild-type D. magna versus (B) a wild-type D. magna with an atypical eye. (C) Normal compound eye with optic nerves connect to optic lobe and cerebral ganglia. (D) Abnormal compound eye protruded from the carapace with irregular shape and arrangement of the ommatidia. The optic lobe is absent in this D. magna. (E) Single layer of gut epithelial cells in the wild-type D. magna versus (F) gut lining with protruding and sloughing of epithelial cells in D. magna with atypical eye. (TIFF) [file pone.0313389.s004.tiff]
